# Evaluation of Anatomical and Functional Outcome of Epiretinal Membrane Surgery in Idiopathic Epiretinal Membrane Patients

**DOI:** 10.7759/cureus.34538

**Published:** 2023-02-02

**Authors:** Qi Zhe Ngoo, Tinesh Thamotaran, Azhany Yaakub, Zamri Noordin, Jane Foo Mei Li

**Affiliations:** 1 Department of Ophthalmology and Visual Science, School of Medical Sciences, Universiti Sains Malaysia, Kota Bahru, MYS; 2 Department of Ophthalmology, Hospital Raja Perempuan Zainab Il, Kota Bahru, MYS; 3 Department of Ophthalmology, Hospital Raja Permaisuri Bainun, Ipoh, MYS

**Keywords:** logmar best-corrected visual acuity, disorganized retinal inner layer, central subfield mean thickness, ellipsoid zone integrity, ilm peel, epiretinal membrane

## Abstract

Objective

To evaluate the anatomical and functional outcomes of an idiopathic epiretinal membrane (ERM) between the observation group and intervention group at six months postoperative.

Design

Prospective cohort study.

Participants

Patients who met the clinical diagnosis of idiopathic ERM in the age frame of 18-80 years; patients with reduced visual acuity (VA), with best corrected VA of 0.2 LogMar or worse, with symptoms of significant metamorphopsia, who visited our center from June 2021 to June 2022.

Methods

All idiopathic ERM patients who fulfilled the inclusion criteria were selected. The data recorded included the year of ERM diagnosis, duration of symptoms, age at diagnosis, gender, ethnicity, and presence of other ocular pathologies. Corrected VA, lens status, ERM configuration, and central subfield mean thickness (CST) in spectral domain-optical coherence tomography (SD-OCT), ellipsoid zone integrity (EZ), and disorganized retinal inner layer (DRIL) were recorded for all patients at diagnosis, as well as 3 and 6 months after diagnosis for non-operated patients. For patients who underwent surgery (pars plana vitrectomy (PPV), internal limiting membrane (ILM), and ERM peel), data were recorded similarly with additional data on the type of surgery (vitrectomy or combined phaco vitrectomy) and the development of intra or post-surgical complications.

Patients receive information on the symptoms associated with ERM, treatment options, and disease progression. After counseling, the patient makes informed consent to the treatment plan. Patients are seen in the 3rd and 6th month from diagnosis. Combined phaco vitrectomy is performed if there is also significant lens opacity.

Main outcome measures

VA, CST, EZ, and DRIL at diagnosis and 6 months.

Results

Sixty subjects (30 interventional and 30 observational arms) were recruited for this study. The mean age in the intervention and observation groups was 62.70 and 64.10 years, respectively. Most ERM patients were female in the intervention group compared to males with 55.2% and 45.2% respectively.

The mean pre-op CST was 410.03 μm in the intervention group compared to the pre-op CST 357.13 μm observation group. There were significantly different among groups in pre-op CST (p=0.009) using the independent T-test. Furthermore, the mean difference and 95% confidence interval in post-op CST were -69.67 (-99.17, -40.17). There were significant differences among groups in post-op CST (p<0.001) using the independent T-test.

Meanwhile, there is no significant association of DRIL between both groups (p=0.23), with 95% CI of mean difference (-0.13, -0.01) using repeated measure analysis of variance (ANOVA) test. There was a significant association of EZ integrity between groups (p=<0.001), 95% CI of mean difference: (-0.13, -0.01) using a repeated measure ANOVA test. Furthermore, the mean post-op VA between pre and post-op VA was significantly different (p<0.001), with a 95% CI of mean difference (-0.85, -0.28). Finally, there is a significant factor association between the duration of ERM and post-op VA (b=.023, 95% CI .001, .05, p<0.05) with our patients.

Conclusion

ERM surgery has shown positive outcomes on anatomical and functional aspects with minimal safety-related risks. It is evident that a longer duration of ERM does give a minimal impact on the outcome. SD-OCT biomarkers, such as CST, EZ, and DRIL, can be used as reliable prognosticators in decision-making for surgical intervention.

## Introduction

Epiretinal membrane, commonly called ERM, is a sheet-like fibrous material that develops on the surface of the inner retina. This disease was identified in 1865 [1]. ERM commonly involves the elderly age group and can be classified into primary or secondary due to various ocular conditions [2,3]. ERM disease progression can be classified into three: regress, stable, and progression. Only about 10-25% of cases have impaired visual acuity (VA) and those with VA worse than 20/200 account for less than 5% [3-5]. They also complain of reduced VA, metamorphopsia, micropsia, or even monocular diplopia [6]. Due to the slow progression, it is common for patients to be observed for elongated periods. Surgical intervention is usually advised after the condition has been monitored, most frequently when patients develop more significant progressive visual blurring with or without metamorphopsia.

ERM removal gold standard treatment is via surgery. Pars plana vitrectomy (PPV) with membrane peeling has been the mainstay surgical treatment since 1978 [7]. Prediction of visual outcomes is essential for patient counseling and for weighing the risks and benefits of surgery. Surgical indications have not been standardized, and therefore clinical results may vary considerably [8,9]. The typical classical indication of surgery is usually the decrease of VA of LogMar 0.3 or worse [10]. The presence of metamorphopsia tends to accelerate surgical intervention. With recent developments in surgery and imaging techniques, there are possibilities for earlier intervention.

Given all of these, what will be the best current approach for patients with a diagnosis of ERM? This study aims to investigate the anatomical and functional outcome of the surgery compared to the observational group. A comparison was also made to identify the prognosticating factors such as central subfield mean thickness (CST), ellipsoid zone integrity (EZ), and disorganized retinal inner layer (DRIL) reliability as well as the impact of the duration of disease toward the outcome of the surgery.

## Materials and methods

A prospective cohort study was conducted involving 60 eyes (30 interventional and 30 observational) from June 2021-June 2022 at Hospital Raja Permaisuri Bainun, Ipoh, and Hospital Raja Perempuan Zainab II, Kota Bharu. All idiopathic epiretinal membrane patients, aged between 18-80 years, with the best-corrected visual acuity (BCVA) of 0.2 or worse LogMar, with symptoms of significant metamorphopsia and reduced visual acuity who visited our center from June 2021-June 2022, were selected for our study. The data that were recorded for all patients included the year of ERM diagnosis, duration of symptoms, age at diagnosis, gender, ethnicity, and presence of other ocular pathologies. Corrected visual acuity, lens status, ERM configuration, and central subfield mean thickness in spectral domain-optical coherence tomography (SD-OCT) were recorded for all patients at diagnosis, as well as three and six months after diagnosis for non-operated patients. The data collection sheet is present in the Appendices.

This study received ethical approval from the Research and Ethical Committee, School of Medical Sciences, Universiti Sains Malaysia (USM/JEPeM/21020197) and the Medical Research & Ethics Committee Ministry of Health Malaysian (NMRR-20-3176-57748, and it was conducted in accordance with the World Medical Association Declaration of Helsinki's ethical principles for medical research involving human subjects.

For those patients who underwent surgery (PPV, internal limiting membrane (ILM), and ERM peel), the same data were recorded similarly at the time of diagnosis and post-surgery at three months and six months. Additional data recorded were the type of surgery (vitrectomy or combined phaco vitrectomy) and the development of intra- or postsurgical complications. However, no minimum follow-up was required for patients who had undergone vitrectomy; this decision was taken in order not to miss patients who might have had a negative visual outcome and might have decided to consult elsewhere. There is no definitive consensus on the classification of ERMs: several groups have proposed different classifications [11,12]. Since we are not studying the morphology of the ERM per se, we have adapted the conventional classification of ERM using the Gass classification of Grade 0, Grade 1, and Grade 2.

The following guidelines are followed in our center for patients with an ERM: if the best corrected visual acuity is equal or worse than 0.2 (LogMar) and symptomatic (poor vision or significant metamorphopsia) at diagnosis, surgical intervention was recommended. Patients receive information on the symptoms associated with ERM, treatment options, and disease progression. After counseling, the patient makes an informed consent on the treatment plan either observation or surgical intervention. Patients are seen in the third month and sixth month from diagnosis. Combined phaco vitrectomy is performed if the ERM is deemed to be the main cause of visual loss but there is also significant lens opacity.

In our centers, a three-port pars plana vitrectomy is performed under retrobulbar anesthesia, with 25G instruments. Two surgeons (JFL and ZN) conducted a complete vitrectomy and indentation to evaluate the retinal periphery. Any tears detected are treated with a laser. After posterior hyaloid removal, the ERM and ILM are stained with dual blue. The ERM is grasped and peeled with end-gripping forceps. If necessary, the ILM is re-stained to improve visualization prior to removal. If phaco vitrectomy is scheduled, lens surgery is performed as the first step of surgery. Postoperatively, patients are treated with topical antibiotics for one week and corticosteroids tapered during the first month. The patients under the observational category were seen at three months and six months with repeat VA, anterior and posterior assessment, and OCT macula.

Statistical analysis was performed with the SPSS program (version 20.0, IBM Corp., Armonk, NY). For categorical variables, number (n) and percentage (%) are presented. For continuous variables, mean, standard deviation (SD), and range are provided. For comparisons between groups, repeated measure analysis of variance (ANOVA) and the independent t-test were used. The simple linear regression test and Spearmen's correlation were used for comparison within groups. A p-value of <0.05 was considered statistically significant.

## Results

Demographics data

A total of 60 subjects (30 interventional and 30 observational arms) were recruited for this study. The mean age in the intervention group was 62.70 years while the mean age in the observation group was 64.10 years. Most of the ERM patients were female in the intervention group compared to males with 55.2% and 45.2%, respectively. Malays led the cases of ERM patients with 62.5% followed by the Chinese and Indians in the intervention group (Table 1).

**Table 1:** Demographic and visual function between observational and intervention groups of idiopathic epiretinal membrane (n=60) ^a^p <0.05 is considered statistically significant based on the independent t-test. ^b^p <0.05 is considered statistically significant based on the Pearson chi-square test.

Variables		Observation= 30	Intervention= 30	p-value
Age (year) (Mean + SD)		64.1 ± 7.4	62.70 ± 8.44	0.656^a^
Sex (n, %)	Male	17 ± 54.8	14 ± 45.2	0.650^b^
	Female	13 ± ​​​44.8	16 ± 55.2	
Ethnicity (n, %)	Malay	9 ±​​​​​​​ 37.5	15 ± ​​​​​​​62.5	
	Chinese	17 ± 65.4	9 ± 34.6	
	Indian	4 ± 40.0	6 ± 60.8	

The anatomical and functional outcome between observational and interventional groups

As stated in Table 2, the mean at diagnosis CST was 410.03 μm in the intervention group compared to the pre-op CST 357.13 μm observation group. There were significantly different among groups in pre-op CST (p=0.009) using the independent t-test. Furthermore, the mean difference and 95% confidence interval after six months CST were -69.67 (-99.17, -40.17), respectively. There were significantly different among groups after six months CST (p<0.001) using the independent t-test.

**Table 2:** Comparison of anatomical outcomes for CST between the observation and intervention groups (n=60) CST= Central Mean Subfield Thickness ^a^Independent t-test, p <0.05 is considered statistically significant

Variable		Intervention Mean (SD)	Observational Mean (SD)	Mean Different (95% CI)	p-value
CST (μm)	At diagnosis CST	410.03 (89.06)	357.13 (59.57)	52.90 (13.74,92.06)	0.009^a^
	After 6 months CST	289.87 (59.46)	359.53 (54.59)	-69.67 (-99.17, -40.17)	<0.001^a^

**Figure 1:**
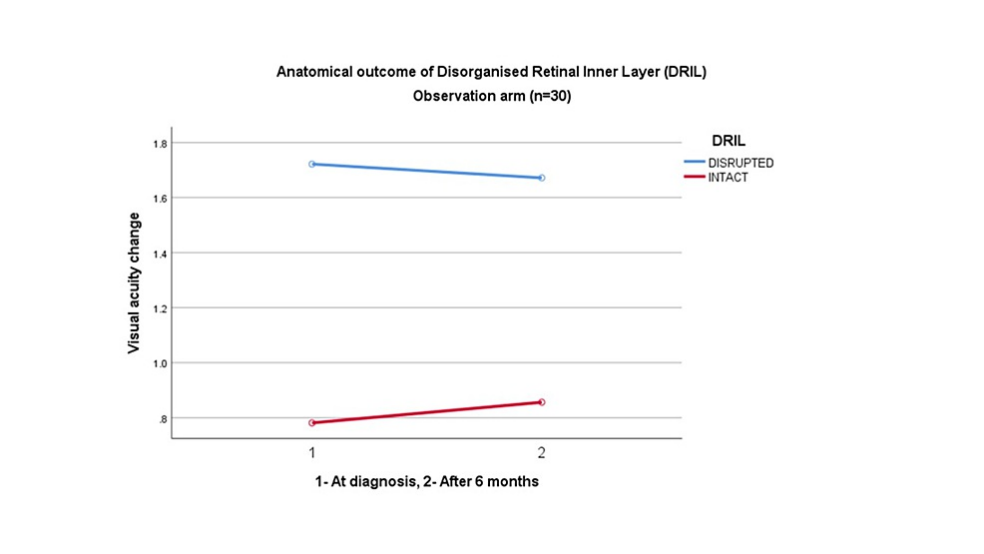
Anatomical outcome of DRIL in the observation arm (n=30) DRIL = Disorganised Retinal Inner Layer

**Figure 2:**
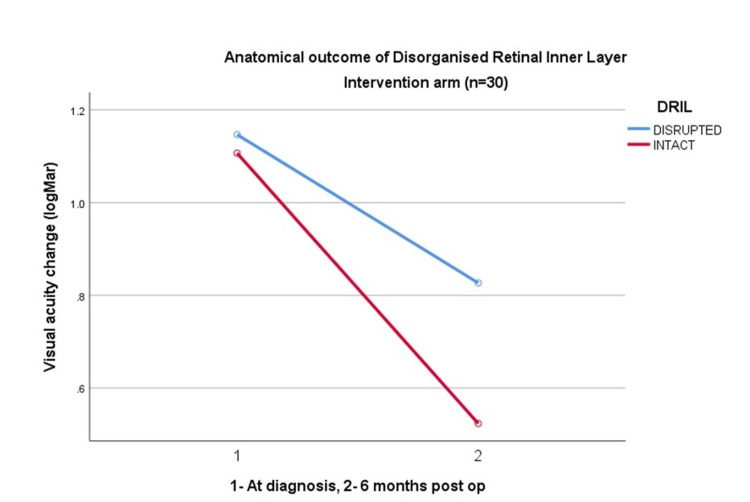
Anatomical outcome of DRIL in the intervention arm (n=30) DRIL = Disorganised Retinal Inner Layer VA = Visual Acuity

Meanwhile, there is no significant statistical association of disorganized inner retinal nerve fiber layer between both groups (p=0.23), with a 95% CI of mean difference (-0.13, -0.01) despite showing improvement in visual acuity seen in the intact DRIL group (Figures 1, 2; Table 3).

**Table 3:** Comparison of anatomical outcome for DRIL between the observational and intervention groups (n=60) DRIL= Disorganised Retinal Inner Layer; ANOVA: Analysis of Variance Repeated measure ANOVA, Significant results (p-value < 0.05)

Variable	Grade	Intervention Mean (SD)	Observational Mean (SD)	Mean Different (95% CI)	p-value
DRIL	Disrupted	1.72(0.13)	1.67(0.13)	0.05 (-0.13, -0.01)	0.23
	Intact	0.78(0.12)	0.86(0.12)	0.08 (-0.15, -0.01)	0.06

There was a significant association of ellipsoid zone integrity between groups (p=<0.001), 95% CI of mean difference (-0.13, -0.01) using a repeated measures ANOVA test (Table 4).

**Table 4:** Comparison of anatomical outcomes for EZ between the observational and intervention groups (n=60) EZ = Ellipsoid Zone Integrity; ANOVA: Analysis of Variance Repeated measure ANOVA, Significant results (p-value < 0.05)

Variable	Grade	Intervention Mean (SD)	Observational Mean (SD)	Mean Different (95% CI)	p-value
EZ Integrity	Disrupted	1.42(0.18)	1.14(0.12)	0.28 (-0.06, -0.63)	0.11
	Intact	1.00(0.12)	0.48(0.10)	0.52 (-0.13, -0.01)	<0.001

On the other hand, the mean post-op visual function between pre and post-op VA is significantly different (p<0.001), with 95% CI of mean difference (-0.85, -0.28). Statistically, there is 95% certainty that the mean difference in post-op visual function among ERM patients lies between -0.85 and -0.28 (Table 5).

**Table 5:** Comparison of LogMar charts between the observation and intervention groups (n=60) VA = Visual Acuity ^a^Independent T-test, p <0.05 is considered statistically significant

Variable		Intervention Mean (SD)	Observation Mean (SD)	Mean Different (95% CI)	p-value^a^
LogMar Visual Acuity	Pre-Op VA	1.13(0.55)	1.22(0.68)	-0.093 (-0.41, 0.23)	0.562
	Post-Op VA	0.67(0.47)	1.24(0.63)	-0.56 (-0.85, -0.28)	<0.001
	VA Change	-0.46(0.53)	0.17(0.16)	-0.16 (-0.68, -0.27)	<0.001

Association between duration of ERM and visual outcome of the ERM surgery

**Figure 3:**
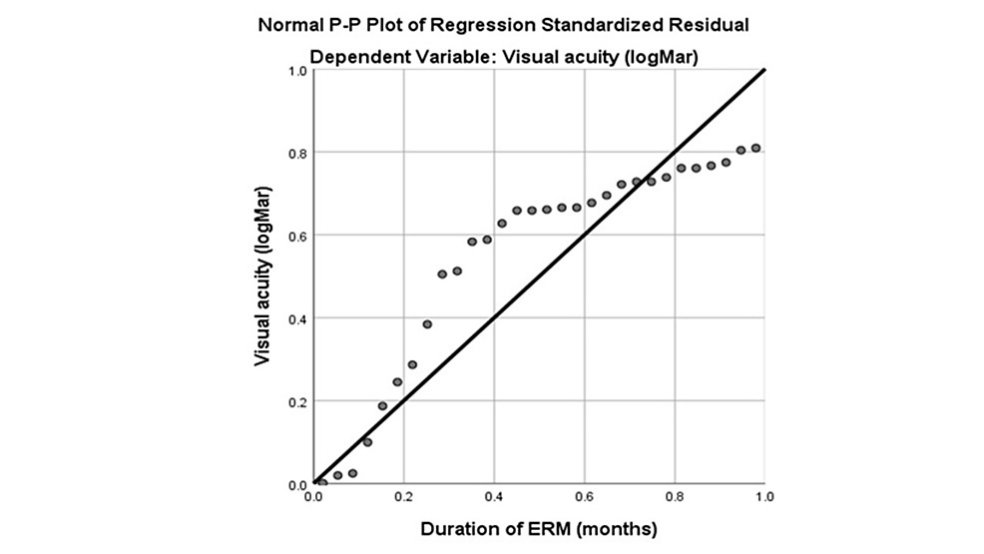
Normal P-P plot of regression VA = Visual Acuity; ERM = Epiretinal Membrane

Based on a simple linear regression test, there is a significant factor association between duration ERM and post-op visual function (b=.023, 95% CI .001, .05, p=0.045) among idiopathic ERM patients with every one-point increase in the duration of ERM, there will be an increase of 0.03-unit of visual acuity (Figure 3; Table 6).

**Table 6:** Comparison of outcomes of the surgical intervention group in terms of duration of ERM (n=30) ERM = Epiretinal Membrane ^a^Simple linear regression R²adjusted=.136, CI = confidence interval; B. p <0.05 is considered statistically significant.

Variable	B	95% CI	β	t	p^a^
Constant	-0.459	[-1.27, -0.425]		-4.107	0.000
Duration of ERM	0.023	[0.001, 0.05]	0.369	2.099	0.045*

## Discussion

The goal of this study is to identify the efficacy of the outcome of both conservative and interventional management. Second, it gives surgeons an advantage in identifying and risk stratifying and choosing a treatment plan for idiopathic epiretinal membrane patients since the demand for the surgery has gone higher in the past few decades. Analyzing these objectives also gives a great advantage to patients to participate in decision-making on choosing a treatment plan. The epiretinal membrane mainly affects the elderly evident in this study. In line with many population-based studies such as the Singapore Indian Eye Study [13] and the Melbourne Collaborative Cohort Study [14], the incidence of the idiopathic epiretinal membrane mainly above 50 years old; however, the highest rate of occurrence is about 70 years to 80 years. In our study, all our patients are above 50 years old with 45% comprising above 70 years old, 35% above 60 years old, and 20% above 50 years old. This evidently supports that increasing age is a strong risk factor associated with the occurrence of ERM [13,15].

Macular thickness, intended also as central subfoveal mean thickness (CST) and average macular thickness, has been widely investigated as a prognostic factor in idiopathic ERM surgery, but the results are not definite. Spontaneous separation of ERM from the retina is known to occur, albeit rarely. However, there were cases of spontaneous separation ERM, which lead to an improvement in VA [16]. In our observation, unfortunately, there was no spontaneous improvement in CST as well as a VA change in the observation arm compared with the intervention arm. There was a significant change in the improvement of CST in the intervention arm with no worsening. We can deduce that spontaneous separation is very rare, especially in the elderly age group [17]. It is widely known that ERM frequently associates with posterior vitreous detachment (PVD) [18]. However, unfortunately, we could not verify all patient's vitreous conditions and the incidence of PVD in this study. Therefore, we could not find an association between the status of PVD to the outcome of CST in our observational group.

The ellipsoid zone (EZ) represents the inner/outer (IS/OS) segments junction of photoreceptors. The alignment of the discs is vital for the normal functioning of the photoreceptors [19]. Hence, the presence of a normal IS/OS junction on OCT images indicates normally functioning photoreceptors [19]. This zone at OCT is strongly associated with functional status and is a valuable prognostic factor for VA after surgery. In our study, we deduced that there is a significant impact on the status of EZ prior to surgery. The majority of our patients with intact EZ had shown better postoperative VA and vice versa for disrupted EZ. They also displayed higher gain in visual acuity in the intact EZ group [19]. In our observation group, disrupted EZ is related to poorer visual acuity [20]. Niwa et al. studied focal macular electroretinogram effects by ERM surgery [21]. They concluded that preoperative a-wave amplitude correlated significantly with postoperative VA. This is consistent with our results that the pre-operative EZ correlated significantly with postoperative VA because the a-wave originates mainly from the combined activity of the photoreceptor and off-bipolar cells [22]. Two out of 21 patients with intact EZ who underwent ERM surgery did not achieve any improvement in visual acuity. This could be due to the slow recovery of photoreceptors. Few studies have reported that a minimum time of around 12 months is needed to allow the photoreceptors to recover eventually to benefit from visual acuity gain after ERM surgery [23]. All these findings suggest that OCT images can provide information on the foveal photoreceptor layer and visual prognosis in eyes undergoing ERM surgery by use of the EZ line.

The disorganized retinal inner layer (DRIL) is the inability to visualize the boundaries of the inner retinal layers, which indicates anatomical damage leading to impairment in the visual transmission pathway. A defective second neuron in the visual system may indicate that the DRIL disrupts pathways that transmit visual information from the photoreceptors to the ganglion cells. Despite being a novice and robust biomarker for diabetic macula edema (DME), its significance is yet to be proven for ERM [24]. Zur D et al. stated that severe DRIL has shown the worst improvement in VA postoperatively in ERM surgery [25]. However, DRIL does not show any significant effect in mild DRIL and is unable to find a correlation between EZ and severe DRIL with VA outcome. We observed in our study that improvement is seen in intact DRIL, however, no statistical significance can be obtained. We predict that this could be due to a few factors such as the involvement of other carrying factors like EZ and/or the small sample size.

The functional outcome that was measured in this study is VA as the parameter. Comparing intervention and observation, we learned that there is a significant difference whereby intervention brings a better outcome compared to observation. Furthermore, we agree with the findings of Dawson et al. [26], highlighting that poorer preoperative VA is associated with greater BCVA change. Thus, baseline BCVA plays a vital role in predicting the extent of visual improvement [27].

There is conflicting evidence regarding the timing of surgery for ERM. Studies have shown promising results for early ERM surgery with better visual outcomes [28]. Cataract formation has been shown to decrease VA three to 12 months after vitrectomy surgery, with a decrease in visual acuity each year [28]. Therefore, the analysis of ERM surgery with the outcome is disrupted by the formation of cataracts. In our study, we have eliminated the probable cause by performing phaco-vitrectomy for all our patients. The results that we obtain after excluding the possible affecting factors are that there is a significant linear relationship between the duration of symptoms and postoperative visual acuity.

Based on our findings, we recommend early intervention in symptomatic epiretinal membrane patients may help in benefiting good postoperative visual outcomes. ERM surgery indefinitely produces good postoperative functional outcomes of visual acuity and anatomical outcome of CST. Biomarkers such as EZ can be used confidently as a prognosticating tool.

## Conclusions

The efficacy of ERM surgery has been proven by multiple studies due to its positive outcomes on anatomical and functional aspects with minimal safety-related risks. It is safe to say that a longer duration of ERM does give a minimal impact on the outcome of the surgery. Utilizing SD-OCT for biomarkers such as CST, EZ, and DRIL in decision-making for surgical intervention has proven to be a reliable prognosticator. In general, ERM surgery carries very minimal safety-related complications.

## Figures and Tables

**Figure 4:**
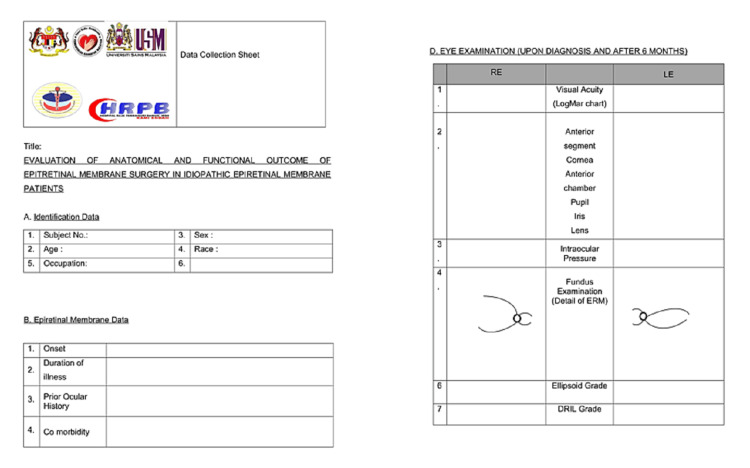
Data collection sheet
